# MetaGOmics: A Web-Based Tool for Peptide-Centric Functional and Taxonomic Analysis of Metaproteomics Data

**DOI:** 10.3390/proteomes6010002

**Published:** 2017-12-27

**Authors:** Michael Riffle, Damon H. May, Emma Timmins-Schiffman, Molly P. Mikan, Daniel Jaschob, William Stafford Noble, Brook L. Nunn

**Affiliations:** 1 Department of Genome Sciences, University of Washington, Seattle, WA 98195, USA; damonmay@uw.edu (D.H.M.); emmats@u.washington.edu (E.T.-S.); william-noble@uw.edu (W.S.N.); 2 Department of Biochemistry, University of Washington, Seattle, WA 98195, USA; djaschob@uw.edu; 3 Department of Ocean, Earth, and Atmospheric Sciences, Old Dominion University, Norfolk, VA 23529, USA; mmika003@odu.edu

**Keywords:** metaproteomics, proteomics, bioinformatics, software, data visualization, mass spectrometry, gene ontology

## Abstract

Metaproteomics is the characterization of all proteins being expressed by a community of organisms in a complex biological sample at a single point in time. Applications of metaproteomics range from the comparative analysis of environmental samples (such as ocean water and soil) to microbiome data from multicellular organisms (such as the human gut). Metaproteomics research is often focused on the quantitative functional makeup of the metaproteome and which organisms are making those proteins. That is: What are the functions of the currently expressed proteins? How much of the metaproteome is associated with those functions? And, which microorganisms are expressing the proteins that perform those functions? However, traditional protein-centric functional analysis is greatly complicated by the large size, redundancy, and lack of biological annotations for the protein sequences in the database used to search the data. To help address these issues, we have developed an algorithm and web application (dubbed “MetaGOmics”) that automates the quantitative functional (using Gene Ontology) and taxonomic analysis of metaproteomics data and subsequent visualization of the results. MetaGOmics is designed to overcome the shortcomings of traditional proteomics analysis when used with metaproteomics data. It is easy to use, requires minimal input, and fully automates most steps of the analysis—including comparing the functional makeup between samples. MetaGOmics is freely available at https://www.yeastrc.org/metagomics/.

## 1. Introduction

Recent years have seen tremendous advancements in the availability of high-throughput “omics” technologies for characterizing complex biological samples. These advancements have fueled the growth of meta-omics as a field for characterizing the metagenomes, metatranscriptomes, meta-metabolomes, and metaproteomes of environmental and microbiome samples comprising a taxonomically diverse (often uncharacterized) community of organisms [1,2,3]. Metagenomics examines questions related to taxonomic composition and genomic architecture of organisms in the sample [4,5]. Meta-metabolomics examines which metabolites are being produced and how those change in response to environmental factors [6]. Meta-transcriptomics aims to use gene expression of mRNA transcripts to track taxonomic and functional abundance [7]. However, transcript and actual protein levels can be poorly correlated because of codon bias, differing rates of protein turnover, and other factors [8,9]. Metaproteomics aims to overcome this by assaying the protein composition directly in order to characterize the taxonomic and functional makeup of a sample at the time of collection [10].

Typically, metaproteomics is carried out by bottom-up, or shotgun, proteomics. Environmental or microbiome samples are digested into peptides (usually with trypsin), the peptides separated by a liquid chromatography column, and then analyzed by inline tandem mass spectrometry. Because these peptides are ionizable and fragment in a predictable manner when using Collision Induced Dissociation (CID), the mass spectrum that results can be interpreted and the peptide sequence can be resolved. The identification of the peptide sequence relies on software (e.g., Comet [11], Sequest [12], Mascot [13], and X! Tandem [14]) that searches the masses of candidate ions against a sequence database. This sequence database may comprise gene products predicted by a metagenomic analysis, annotated reference proteomes of organisms likely to be present (e.g., sequences from NCBI nr [15] for human or specific bacteria), or a combination of the two [16,17]. Lists of tens of 1000’s of peptides are generated in a single 60 minute mass spectrometry analysis. The identified peptides are used to predict which proteins from the sequence database are present in the sample [18,19]. Once a final protein list is predicted, the relative abundance of proteins may be estimated using spectral counting, which counts the number of times peptides that matched each protein were observed by the mass spectrometer. Spectral counts are then normalized using one of various methods [20,21,22,23]. For example, the Normalized Spectrum Abundance Factor (NSAF) [22] adjusts the spectral counts based on the total number of proteins identified in the sample and the respective lengths of those proteins. Finally, the normalized values, functional annotations and taxonomic assignments for the predicted protein lists are used to ascribe relative abundances to functions and taxa and these abundances are compared between samples.

However, metaproteomics datasets present unique challenges for which traditional protein inference and spectral counting methods, such as NSAF, were not designed. From the list of peptides, the standard protein inference methods predict which proteins are present by considering all identified peptides and correlating them back to the protein sequence database used to search the data. Parsimonious protein inference follows the Occam’s razor principle, (e.g., if protein A or B can explain the occurrence of 3 peptides but protein B can also explain the occurrence of 6 other peptides, then it is probably true that protein B is present, not A) [24,25,26]. As a result of this simplification, protein inference is contentious within the mass spectrometry community, even for single organism studies [27,28]. In the case of microbiomes, this issue becomes far more complex because there are hundreds, possibly thousands, of species contributing to the peptides present. In many cases, when searching mass spectrometry data from microbiomes against a site-specific metaproteome database, a single peptide sequence might be found in more than 1000 of the proteins in that database. Upon close examination, it becomes apparent that that peptide, in many cases, is present in proteins that come from a wide variety of species. So the question becomes, how do you report a protein’s presence when the peptide evidence suggests it is from 1000 different species? Deciding to include all proteins matched by peptides will almost certainly include too many and selecting only one to report has inherent bias. Further, because NSAF uses the final list of proteins to normalize the NSAF values for each protein, erroneously including too many or too few proteins may have a significant impact on these values. And, in the case where public databases are used in lieu of metagenomic-derived sequence databases, peptides are matched to proteins that may not be in the sample at all [16,17]. This further confounds NSAF, since the lengths of the proteins in the database are used to normalize the abundance value and the lengths of the protein sequences in the sample may differ from the lengths of proteins in the database. Given that (1) the mass spectrometry assay identifies peptides rather than proteins; (2) we are interested in characterization of functions and taxa rather than proteins; and (3) the list of inferred proteins may be unreliable, we contend that protein inference should be avoided altogether. Instead we pursue a peptide-centric approach.

Several tools for functional and taxonomic analysis of metaproteomes are currently used by the metaproteomics community. Though MEtaGenome ANalyzer, or MEGAN [29], was designed for metagenomics data, it can be used for metaproteomics analysis by substituting the expected genomic BLAST with protein BLAST. The visualization tools for MEGAN rely on RefSeq (NCBI) annotations and were designed for metagenomics data and utilizes full sequence information, such as an assembled gene or gene product (i.e., protein). MetaProteomeAnalyzer (MPA) [30] is a complete metaproteomics pipeline spanning initial database search through final visualization. To deal with the issues associated with protein inference-based analysis of function and taxonomy, MPA collapses highly-redundant protein hits into “metaproteins”, whose annotations are derived from its component proteins. MPA then quantifies taxa and functions using spectral counting. Some features of MPA also assume UniProtKB was used to match spectra to peptides, and custom metagenome-derived databases may not be natively supported. Unipept [31], a web-based, peptide-centric metaproteomics application, provides advanced and beautiful data visualization tools but is currently limited to taxonomic analysis and cannot analyze peptides that are not present in UniProtKB.

The web-based MetaGOmics tool is designed to perform functional and taxonomic analysis without the need to predict which proteins are present in the sample. MetaGOmics works at the peptide level, by assigning functional and taxonomic annotations to each peptide based on the annotations of proteins matched in the sequence database used to search the data. Then the relative abundance for each of those annotations is incremented by the relative abundance for that peptide. In the case of spectral counting, this ensures each spectrum increases the spectral count for each functional or taxonomic annotation only once, which may not be the case if we assumed each protein matched by the peptide was in the sample. The MetaGOmics tool requires minimal input, only the sequence database (as a FASTA file) used to search the data and the list of identified peptide sequences and associated relative abundances (e.g., spectral counts). All subsequent processing, including any necessary sequence homology searching, are performed by the MetaGOmics servers. The web application includes tools to visualize, download, and compare data between experiments, including statistical tools for identifying GO annotations with statistically significant changes. MetaGOmics is open-source (https://github.com/metagomics/, Apache 2.0 license) and freely available to use at https://www.yeastrc.org/metagomics/.

## 2. Methods

### 2.1. Web Application Implementation

MetaGOmics is implemented as a database-backed web-application for submitting and visualizing results and a series of distributed server-side programs to run sequential parts of the pipeline on other servers as needed. The web application was developed using Java, HTML, CSS, SVG, and Javascript; and designed to run on the Apache Tomcat (http://tomcat.apache.org/) Java servlet container and the Struts application framework (http://struts.apache.org/). The relational database was developed using the MySQL (https://www.mysql.com/) relational database management system. The server-side programs are implemented in Java and execution of the programs is managed by the JobCenter [32] platform for managing distributed computational job execution. All source code for both the web application and server-side programs are available at https://github.com/metagomics.

### 2.2. MetaGOmics Algorithm

The MetaGOmics algorithm is designed to be applied to spectrum identifications resulting from standard bottom-up shotgun proteomics analysis of metaproteomics samples. The spectrum identifications may be derived from standard analysis workflows, such as those using Comet, Mascot, X! Tandem, or Sequest. There are no requirements that the protein sequence database comprise entries from any particular public database, and may be made from predictions resulting from, for example, metagenomic sequencing. The algorithm takes as input the list of identified peptides, the relative abundance for each peptide (e.g., spectral count), and the protein sequence database (as a FASTA file) used to search the data. Note that the uploaded FASTA file need only contain the proteins matched by any of the uploaded peptides and we recommend trimming very large protein databases in this way.

**Figure 1:**
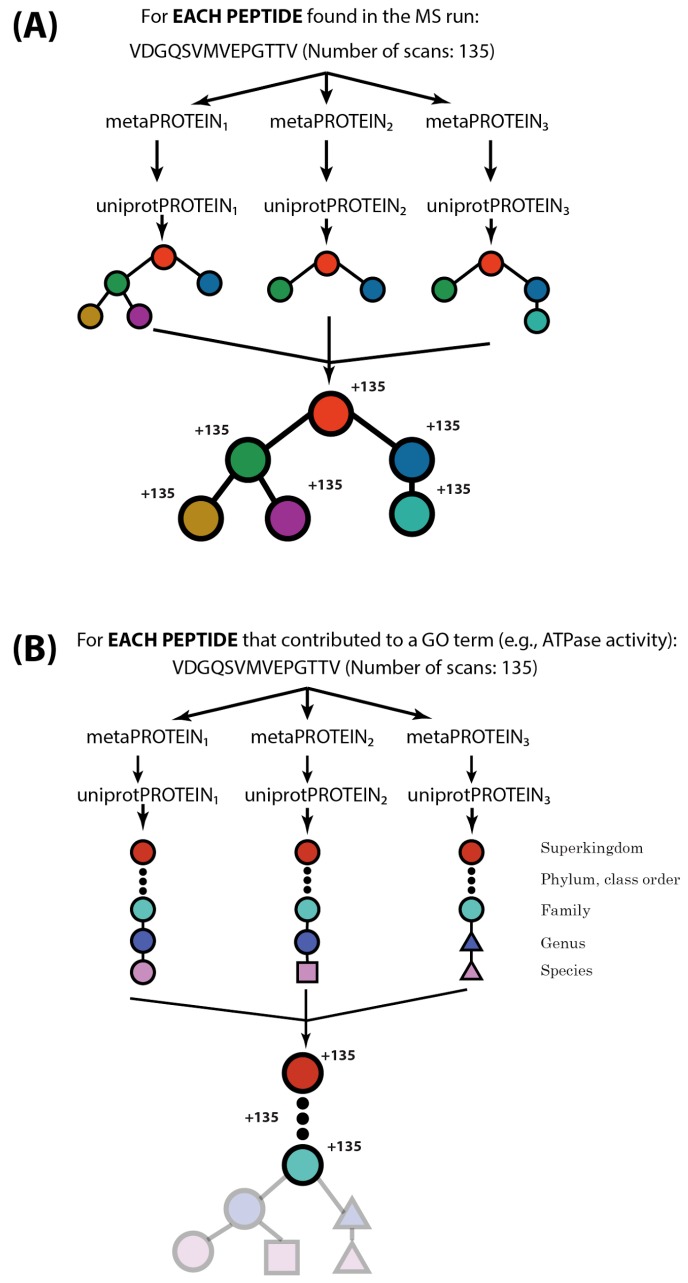
The MetaGOmics algorithm. (**A**) The first phase of the algorithm (functional analysis) examines all peptides identified in a mass spectrometry (MS) experiment. Each peptide is matched to proteins in the FASTA file (metaPROTEIN in figure), those are matched to UniProtKB proteins via BLAST (uniprotPROTEIN in figure), and Gene Ontology (GO) annotations for the UniProtKB proteins are used to create complete GO graphs for each protein containing direct annotations and all ancestor terms. All GO graphs from all proteins matched by a peptide are merged into a single, non-redundant GO graph (the union of the sets), and the spectral count of each term is increased by the spectral count for the peptide. This process is repeated for all peptides in the experiment to obtain final spectral counts for all GO terms; (**B**) The second phase of the algorithm, taxonomic analysis of functions, examines all peptides that are assigned a specific GO term. Each peptide is matched to a FASTA protein (metaPROTEIN in figure), the FASTA proteins are matched to UniProtKB proteins via BLAST (uniprotPROTEIN in figure), and taxonomic annotations for the UniProtKB proteins are used. A taxonomic tree is generated containing the direct taxonomic annotations and all ancestor terms. All taxonomic trees resulting from all matched proteins are merged such that the resulting tree contains only those terms present in all trees (the intersection of the sets). The taxonomic terms have their spectral count increased by the spectral count of the peptide. After all peptides assigned to a GO term are processed, the ratio of the spectral count of each taxonomic term to the total spectral count of the GO term is calculated. This provides the relative, unambiguous contribution (in spectral count) of each taxon to a GO term at any arbitrary level of the taxonomic tree.

The first step of the algorithm estimates the relative abundance of protein functions based on the relative frequencies of peptides that can be ascribed to any protein with those functions (Figure 1a). This is done by first matching peptides to unannotated proteins in the FASTA file, where peptides must be N-terminal in the protein or C-terminal to a lysine or arginine (tryptic digestion is assumed) and leucine and isoleucine are treated interchangeably. Those proteins are then annotated by performing Basic Local Alignment Search Tool (BLAST) [33] on these protein sequences against a large, well-annotated protein sequence database (such as UniProtKB [34]) to find Gene Ontology (GO) [35,36] annotations. Then, for each peptide, a non-redundant directed acyclic graph (DAG) is constructed from all of the GO terms for all of the proteins matched by that peptide. Because of the hierarchical nature of GO, the peptide DAG includes both the GO terms with which proteins were directly annotated as well as all ancestor terms (using the “is a” relationship) to the root of the GO DAG. The spectral count for each GO term in this DAG is then incremented by the spectral count of the peptide that gave rise to it. After all peptides are processed, the final spectral count for each GO term is then normalized by dividing its count by the total number of peptide spectrum matches in the search. A table can be produced containing all GO terms found in the sample, the number of times peptides indicating those GO terms were observed (spectral count), and the proportion of all scans in the experiment that were attributable to that GO term (Table 1).

The second step of the algorithm estimates the relative taxonomic contribution to the number of peptide identifications for each GO term (Figure 1b). For a given GO term (e.g., “calcium ion binding”), this is done by first collating all the peptides that provided evidence for that GO term. Each of these peptides is matched to proteins in the FASTA database, and the UniProtKB BLAST hits are used to provide taxonomic classifications of the proteins. Then for each peptide, the lowest common ancestor (LCA) is found from all proteins matched by this peptide. This is the most specific taxonomic unit for which we can say this peptide provides unambiguous spectral evidence. For example, if the peptide matches proteins that each were found in species belonging to different orders of class Betaproteobacteria, we identify the class Betaproteobacteria as the LCA for which this peptide provides spectral evidence. We then increment the spectral count for the LCA and all of its parent taxa (e.g., Proteobacteria (phylum) and Bacteria (superkingdom) in the case of Betaproteobacteria (class)) by the spectral count for the peptide. After processing all peptides for a given GO term, a table is produced containing (1) all taxa that provided unambiguous evidence for that GO term; (2) the number of times peptides were observed from that taxon for this GO term; and (3) the proportion of all spectra for this GO term that are attributable to that taxon (Table 2).

The third step of the algorithm compares the spectral counts for GO terms between different experiments. This is done by first calculating the log-fold difference (base 2) between the proportions of spectra attributable to a given GO term in two experiments. Because a GO term might only be observed in one of the two experiments and the log-fold change using zero in one of the conditions is undefined, a Laplace correction is performed on the spectral counts for each GO term by adding one to the spectral count for all GO terms and recalculating the proportions. The Laplace correction is equivalent to assigning a uniform prior probability to each GO term. The log-fold change is then calculated between these Laplace-corrected proportions. A *p*-value is calculated using a two-tailed test of proportions that tests the null hypothesis that the proportion for this GO term is the same in the two experiments against the alternative hypothesis that the ratios are different. Multiple hypothesis testing is controlled for using either a Bonferroni correction or a Benjamini-Hochberg adjustment [37,38] (Table 3). At this time, MetaGOmics only supports pairwise comparisons between two experiments, though the data from multiple experiments may be downloaded and compared using any preferred analysis method.

### 2.3. Specifying Relative Abundance

The required inputs to use MetaGOmics include the protein database used to search the MS data and a peptide list with associated abundance measures (reported as integers). For the sake of simplicity, this manuscript focuses on relative abundances reported as spectral counts. However, MetaGOmics is not limited to spectral counting. This abundance can just as easily be the total area under the curve from the extracted ion chromatogram for each peptide. MetaGOmics performs its analysis in terms of relative abundances in each sample, not absolute abundances. In the case of areas under the curve, the abundance of a given peptide would be considered as the proportion of its area under the curve to the total areas under the curves for all peptides. It is this proportion that would be compared between samples. Examining the changes in functional and taxonomic abundance between samples in terms of changes in absolute abundance is a current focus of development, and functionality specifically designed to handle this case will be added to the MetaGOmics website soon.

### 2.4. Running BLAST

NCBI BLAST is performed as-needed on behalf of users to find Gene Ontology and taxonomic annotations for proteins in their protein sequence database. Users may select the BLAST database (UniProtKB TrEMBL or Swiss-Prot [39]), E-value cutoff (currently defaults to 1 × 10^−10^), and whether or not to only use the top BLAST hit (currently defaults to true). If only the top BLAST hit is used, then all BLAST hits tied for the best score will be used (if the best score is less than or equal to the E-value cutoff). If not only the top BLAST hit is used, then all hits with an E-value meeting the cutoff are used. At the time of this writing, MetaGOmics runs NCBI BLAST 2.6.0+ on two Linux servers, each with 56-core Intel Xeon E5-2697 CPUs.

### 2.5. Expected Wait Times

The time required to process the initial FASTA upload ranges from a few minutes to a few hours, depending on the size of the FASTA file. Subsequent uploads of the same FASTA file will be instantaneous. To speed up processing, it is highly recommended that this FASTA file be trimmed to include only proteins represented by peptide matches in the results. Please see our GitHub repository for a program to aid in this trimming (https://github.com/metagomics/).

The time required to process uploaded peptide lists and associated spectral counts ranges from a few minutes to more than a day. This time depends on the number of peptides, whether BLAST has already been run on some proteins from this FASTA file from previous analyses, the size of the FASTA file, whether BLAST is searching UniProtKB TrEMBL or Swiss-Prot, and whether or not results beyond the top BLAST hit are being used. Using UniProtKB TrEMBL and retaining more than the top BLAST hit will result in longer processing times. 

In all cases, the expected run times may be significantly impacted by user demand. All requests are processed on a first come, first served basis.

### 2.6. Unknown Gene Ontology Annotations

In the case where no GO annotation is found for a peptide, an “unknown” annotation is created and added as a direct child of the root node of the relevant aspect of the GO DAG. For example, if no annotations were associated with a peptide for the “molecular function” aspect, then an “unknown molecular function” node is added as a child of the “molecular_function” root node. The spectral count for this unknown annotation (and the “molecular_function” root node) is thus increased by the spectral count for that peptide and is included in reported data.

### 2.7. Analysis of Ocean Metaproteomics Dataset

An example MetaGOmics result set was generated by comparing Bering Strait (BSt) surface water (7 m) samples to Chukchi Sea (CS) bottom water (55.5 m) samples. Briefly, microbial fractions were collected on 0.7 µm filters after passing 15 L of seawater through both 10 µm and 1 µm filters to remove larger eukayrotes. These samples were collected, digested, analyzed on a Q-Exactive-HF, and data were searched according to the methods description in May et al. [40].

Separate results files were created for each of the BSt and CS conditions that contained the combined Comet results for the two replicates from each condition. The BSt and CS results were each post-processed using Percolator [41] version 2.10 with the -X option. Lists of identified peptides and spectral counts were parsed from the resulting Percolator XML files using an in-house script, filtering peptide identifications on a *q*-value of 0.01 and then using peptide spectrum matches (PSMs) filtered on a *q*-value of 0.01 for spectral counts. The metaproteome FASTA file (described by May et al.) was filtered using an in-house script to only include protein sequences for which there were peptide matches. Note that this step is not required, but speeds up processing. Both of these scripts are available at our GitHub repository at https://github.com/metagomics/. The resulting list of peptides, associated spectral counts, and filtered FASTA file were uploaded to the MetaGOmics server, with settings to use UniProtKB TrEMBL, an E-value cutoff of 1 × 10^−10^, and only keep the top hit as BLAST settings. The results of the re-analysis are available at https://www.yeastrc.org/metagomics/ocean.

## 3. Results and Discussion

### 3.1. Web Application

**Figure 2:**
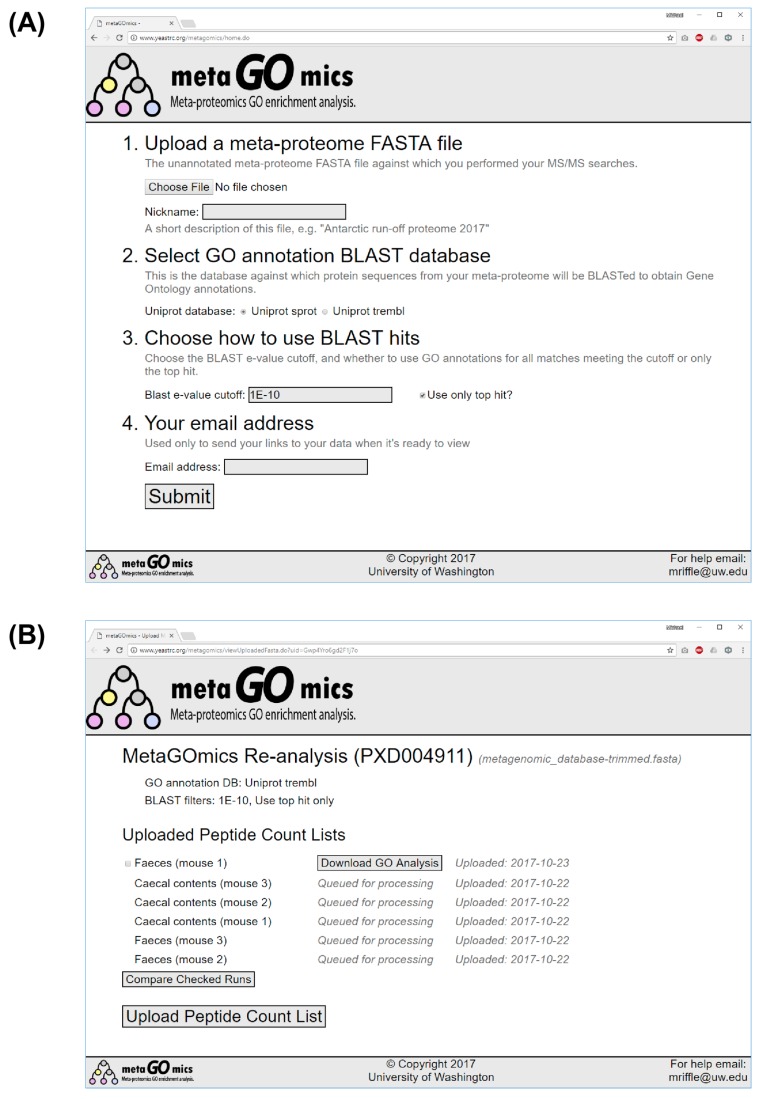
Screenshots from the MetaGOmics web application. (**A**) A user fills out an initial form to create a context for MetaGOmics analysis. The user (1) uploads a FASTA file containing a database of protein sequences to which peptides should be matched, (2) selects a BLAST database to use for protein annotations, (3) chooses cutoffs for the BLAST hits, and (4) enters an email address to be notified when processing is complete; (**B**) After a user submits the form in part (**A**), a unique URL is created for a page where a user may perform MetaGOmics analysis using the desired FASTA file, BLAST database, and BLAST cutoff settings for all uploaded data. To upload data for analysis, the user clicks “Upload Peptide Count List” to upload a text file containing peptide sequences and spectral counts. Each row under “Uploaded Peptide Count Lists” shows each requested analysis and its current status. Upon completion, users may click the “Download GO Analysis” button to download the results as text reports or images. Two analyses may be compared by clicking the checkbox next to two rows and clicking “Compare Checked Runs.” Comparisons may also be downloaded as text reports or as images.

The MetaGOmics web application is available at https://www.yeastrc.org/metagomics/. The user is presented with a simple interface for creating an initial context for the analysis of metaproteomics data (Figure 2a). To create this context the user (1) uploads a FASTA file containing the protein sequences to which peptides identified in any of the experiments are to be matched (e.g., the FASTA file used to search the data); (2) selects a database against which proteins will be searched by BLAST (i.e., UniProtKB/Swiss-Prot or UniProtKB/TrEMBL); (3) chooses which BLAST matches should be considered; and (4) enters an email address where they may be contacted when results are ready.

Upon submitting this form, a unique URL is created where users may request analysis of peptide lists, visualize results, and download text reports from different experiments based on the submitted FASTA file and requested BLAST settings (Figure 2b). This URL contains an unguessable hash string as part of the URL that serves to secure the data. Users will receive an email containing a link to the private URL after submitting the FASTA file. This URL should be retained or recorded. The links do not expire.

To submit data, the user selects “Upload Peptide Count List” and submits a tab-delimited text file containing the peptide list and associated spectral counts from a given experiment. Submitting this file initiates the sequential running of multiple server-side programs to perform the analysis. Once the analysis is complete, the user will receive an email notification and another link to the private URL, which allows for downloading and visualizing the result of this analysis. Users may submit the results of as many experiments as they would like to be analyzed using this FASTA file and BLAST settings, such as the results of each condition or replicates.

To view the results, users click the “Download GO Analysis” button next to the nickname they gave a set of results. This opens an overlay where users may choose to download the results as a text report or as an image of the GO DAG. The text report contains the GO term accession number, GO aspect, GO name, spectral count, and proportion of all spectra in the experiment for each GO term found in the experiment (spectral ratio). The image contains a graphical representation of the GO terms in their hierarchical structure, labeled with their spectral count, spectral ratio, and shaded according to the spectral ratio. The images may be downloaded in portable network graphics (PNG) or scalable vector graphics (SVG) formats.

**Figure 3:**
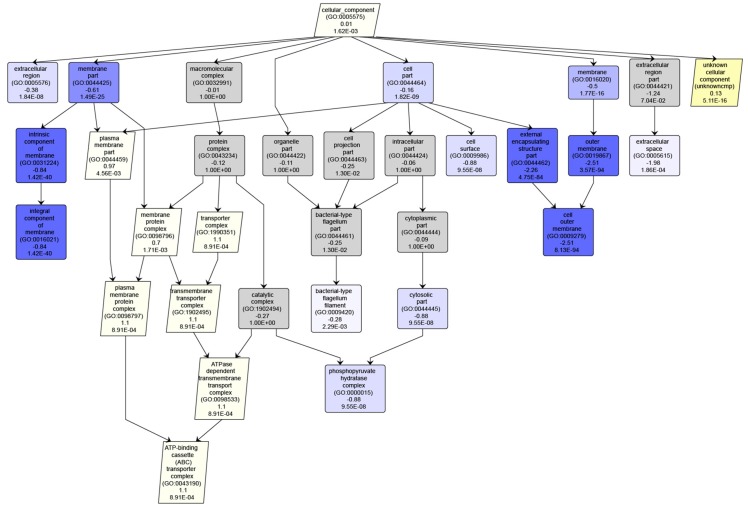
An example GO graph generated by comparing two experiments using the MetaGOmics server. Each node is a GO term, with lines indicating edges between those nodes in the GO structure. Each node is labeled with the name of the GO term, the log fold change, and the *q*-value. Nodes with a positive log-fold change are shaped as parallelograms, and shaded yellow—where darker shades of yellow indicate more significant *q*-values. Nodes with a negative log-fold change are shaped as rectangles, with shades of blue indicating *q*-value significance. Grey terms are not statistically significant. In this example, GO terms with the “cellular component” aspect were compared. The ratio of spectra in the second experiment matching proteins that localized to the outer cell membrane, extracellular space, phosphopyruvate hydralase complex, and integral component of the membrane were significantly reduced. Whereas, the ratio of spectra matching proteins with an unknown cellular component and ATP-binding (ABC) transporter complex were increased.

If multiple experiments have been analyzed, users may check the box next to any two and click the “Compare Checked Runs” to download a text report or images comparing the ratios of GO terms in the two experiments. The downloaded report includes the GO accession, GO aspect, GO name, ratios from both experiments, PSM counts from both experiments, the log (base 2) fold difference in the ratios, corrected *p*-values, and *q*-values (Benjamini-Hochberg adjustment) for all GO terms found in either experiment. Instead of all GO terms from either experiment, the images contain a “trimmed” depiction of the identified GO DAG that is color coded according to statistical significance (Figure 3). Trimming is accomplished by iteratively removing all leaves from the DAG that have a *q*-value greater than 0.01. The result is a DAG where all leaves have a *q*-value less than or equal to 0.01; note that parents of these nodes may have non-significant *q*-values. This is done to preserve the structure of the DAG, including the relative level of specificity of GO terms in the DAG, while still removing much of the noise of insignificant results. GO terms with higher ratios in the second experiment are shaded yellow, and terms with lower ratios are shaded blue, where the intensity of each color depends on the significance of the *q*-value. Grey terms are not statistically significant. Each GO term is labeled with its name, accession number, log fold change, and *q*-value. The images may be downloaded as PNG or SVG.

### 3.2. Example Analysis: Ocean Metaproteomics

**Figure 4:**
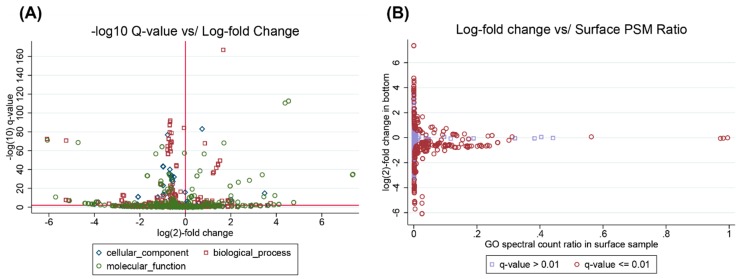
GO term statistics produced by a MetaGOmics analysis comparing ocean surface to bottom water samples from May et al. All data from the analysis are available at https://www.yeastrc.org/metagomics/ocean. (**A**) Volcano plot depicting the negative log (base 10) of the *q*-value versus the log (base 2) fold change for all GO terms found in either sample. A horizontal reference line is added for a *q*-value cutoff of 0.01. A vertical reference line is added for no change. Each point is a GO term and has been colored and re-shaped according to its GO aspect; (**B**) A scatter plot depicting the log (base 2) fold change from the surface sample to the bottom sample for each GO term versus that GO term’s spectral count ratio in the surface sample. Each GO term has been colored either lavender (not statistically significant) or red (*q*-value ≤ 0.01).

An example dataset was generated via a MetaGOmics analysis of the ocean metaproteomics dataset described in May et al. [40] (see methods) (Figure 4). These data compare the relative abundance of peptides attributable to GO terms between Bering Strait surface water (7 m) samples to Chukchi Sea bottom water (55.5 m) samples. A highly-filtered set of results from the analysis is presented in Table 4, where only the statistically significant leaves of the resulting GO DAG are presented. The surface samples have a higher relative abundance of peptides relating to metabolism, translation, GTP utilization, and photosynthesis. Where, the bottom samples have a higher relative abundance of peptides relating to metal binding, protein refolding, DNA repair, and ATP utilization. These results are consistent with what would generally be expected in surface and sea bottom ocean samples. These results may be viewed in their entirety at https://www.yeastrc.org/metagomics/ocean.

### 3.3. Interpreting Results With Many “Unknown” GO Annotations

It is important to pay close attention to the “unknown” GO terms when interpreting changes in relative abundance for GO terms between samples, particularly more general GO terms. A peptide will receive an “unknown” GO annotation for two reasons: there were no BLAST matches for that peptide that met the E-value cutoff or there were no GO annotations for any of the UniProtKB proteins matched via BLAST for the respective GO aspect (molecular function, biological process, or cellular component). In either of these cases, the spectral count of the “unknown” GO term for the respective GO aspect is increased by the spectral count for the peptide. However, it is important to consider that if we did know the true annotations for the unknown peptides, it is likely that many of them would be annotated with a molecular function, biological process, or cellular component that falls under the most general GO terms.

For example, a peptide has a spectral count of 100, but no GO annotation for molecular function can be found. The spectral count for the “unknown molecular function” is increased by 100. However, if the GO annotation were known, it is quite likely it would fall under “binding”, “catalytic activity”, “structural molecule activity”, or some other general term for molecular function. In experiments with a large ratio of unknown molecular functions, it is likely that the ratios of the general GO terms for molecular functions are really higher than reported. As such, interpretations of changes in relative abundance of general GO terms should be treated with care when there is a large ratio of “unknown” GO annotations in one of the experiments.

### 3.4. Interpreting Taxonomic Changes

When a taxonomic tree is calculated for a peptide, only the taxa that are unambiguously matched by that peptide have their spectral counts incremented. For example, if a peptide matches two proteins with the same species, the taxonomic node for that species and all of its ancestors (genus, family, order, and so on to kingdom) have their spectral counts increased by the spectral count for that peptide. If a peptide matches two proteins with different species, but the same genus, we consider that genus to be the most specific taxonomic unit for which this peptide provides unambiguous evidence. Consequently, if a peptide matches multiple proteins in different families, but with the same class, then that class is the most specific taxonomic unit for this peptide. Only the spectral counts for the most specific, shared taxonomic unit for a peptide (and all of its ancestors to the root) are increased by that peptide.

The ramifications of this approach are that more specific levels of the taxonomic tree (i.e., species and genus) may have fewer total spectral counts than more general levels of the taxonomic tree. A peptide that matches a very taxonomically homogeneous set of proteins is able to provide unambiguous evidence for more specific taxa than a peptide that matches a very taxonomically diverse set of proteins. This may be seen in the data by summing the fraction of a GO term’s spectral count that is unambiguously attributable to all the taxa at different levels in the taxonomic tree. For example, when summing the fraction at a general taxonomic level (e.g., order), the fractions associated with all the orders may approach one. This means that nearly all the peptides provided unambiguous taxonomic evidence to at least the order level of the taxonomic tree. On the other hand, if we pick a more granular level (e.g., genus), then the fraction may be considerably lower, such as 0.25. This would indicate that only 25% of the spectra could be unambiguously assigned to a specific genus. Each step up in the taxonomic tree (to a more general term) will have a summed fraction greater than or equal to the step below.

We recommend that when interpreting the taxonomic contributions to a GO term’s spectral count, that the most specific taxonomic level that sums nearly to one be chosen. This is the most specific level of unambiguous taxonomic inference that makes use of most of the spectra.

### 3.5. Current Usage

An early implementation of the functional analysis and experimental comparison steps of the MetaGOmics algorithm (implemented then as in-house scripts) was used to perform a GO-based functional analysis for Timmins-Schiffman, et al. (2017) [17]. This study aimed to characterize the effect of the chosen protein sequence database on the peptide yield and biological inferences that are made from environmental metaproteomics data. It was found that using the metagenome-derived metaproteome yielded a larger number of confident peptide identifications versus protein sequences constructed from existing public databases. It was also found that the choice of sequence database had a profound effect on the functional annotation of the experiment, which could lead to profoundly different biological conclusions.

## 4. Conclusions

As the field of metaproteomics grows, standardized approaches to data analysis must be established to allow comparisons between treatments (e.g., gut microbiomes) or environmental locations (e.g., ocean transects). In addition to the research field having a desire to compare microbiome proteomes between treatments or sites, temporal shifts in community structure and function are paramount to creating accurate models for predicting efficacy of treatments or, in the case of the environment, tracking the effects of global climate change or anthropogenic perturbations. The complexity of peptide sequence assignment in mixed community proteomics cannot be simplified by assuming protein inference yields accurate depictions of the community.

Here we present MetaGOmics, an algorithm and web application for peptide-centric functional and taxonomic analysis of metaproteomics samples, and comparisons between those samples. The MetaGOmics algorithm is designed to overcome drawbacks implicit in a protein-centric approach, in which spectral counting of proteins is used as the basis for functional analysis. Because MetaGOmics requires as input only a list of peptide sequences and associated abundances, the method works with data from any shotgun proteomics pipeline. The web application includes tools for submitting data, viewing results, and downloading reports. MetaGOmics is open-source (https://github.com/metagomics/) and free to use at https://www.yeastrc.org/metagomics/.

## Figures and Tables

**Table 1:** Small subset of GO terms, spectral counts, and relative abundance ratio in a hypothetical mass spectrometry experiment.

GO Accession String	GO Aspect	GO Name	Spectral Count	Ratio
GO:0005575	cellular_component	cellular_component	12,217	1
GO:0008150	biological_process	biological_process	12,217	1
GO:0003674	molecular_function	molecular_function	12,217	1
unknownprc	biological_process	unknown biological process	5472	0.45
GO:0005488	molecular_function	binding	4185	0.34
GO:0097159	molecular_function	organic cyclic compound binding	3579	0.29
GO:1901363	molecular_function	heterocyclic compound binding	3579	0.29
GO:0005524	molecular_function	ATP binding	1712	0.14
GO:1901566	biological_process	organonitrogen compound biosynthetic process	1353	0.11
GO:0042026	biological_process	protein refolding	1145	0.09
GO:1990351	cellular_component	transporter complex	200	0.02

**Table 2:** For a given GO term, the taxa, spectral count, fraction of this GO term’s spectral count, and fraction of all spectra in the experiment that could be unambiguously attributed to each respective taxon. E.g., 88% of the spectra for this GO term were attributable to the Bacteroidetes phylum. 3.5% of the spectra in the experiment were attributable to this GO term and the Bacteriodetes phylum.

Taxon Name	Taxonomy Rank	Spectral Count	Ratio of GO	Ratio of Experiment
*Bacteria*	superkingdom	240	0.88	3.50 × 10^−2^
*Bacteroidia*	class	141	0.52	2.05 × 10^−2^
*Bacteroidetes*	phylum	141	0.52	2.05 × 10^−2^
*Bacteroidales*	order	141	0.52	2.05 × 10^−2^
*Prevotella*	genus	81	0.3	1.18 × 10^−2^
*Prevotellaceae*	family	81	0.3	1.18 × 10^−2^
*Firmicutes*	phylum	41	0.15	5.97 × 10^−3^
*Lactobacillales*	order	33	0.12	4.81 × 10^−3^
*Lactobacillaceae*	family	33	0.12	4.81 × 10^−3^
*Lactobacillus*	genus	33	0.12	4.81 × 10^−3^
*Bacilli*	class	33	0.12	4.81 × 10^−3^
*Prevotella sp. CAG:873*	species	23	0.08	3.35 × 10^−3^
*Clostridiales*	order	6	0.02	8.74 × 10^−4^
*Clostridia*	class	6	0.02	8.74 × 10^−4^
*Actinobacteria*	phylum	5	0.02	7.28 × 10^−4^

**Table 3:** For the comparison of two hypothetical MS experiments, a small subset of the GO terms, log-fold changes, and *q*-values for GO terms detected in the two experiments.

GO Name	Fold Change	*q*-Value
outer membrane	1.55	5.27 × 10^−106^
cell outer membrane	1.55	5.61 × 10^−106^
external encapsulating structure part	1.5	5.64 × 10^−102^
membrane	1.14	3.00 × 10^−101^
receptor activity	1.47	5.03 × 10^−93^
intrinsic component of membrane	1.44	6.37 × 10^−88^
integral component of membrane	1.44	6.37 × 10^−88^
molecular transducer activity	1.35	4.14 × 10^−81^
membrane part	1.01	4.69 × 10^−53^
carbohydrate derivative binding	−2.03	1.25 × 10^−49^
ribonucleotide binding	−2.03	1.25 × 10^−49^
purine ribonucleoside binding	−2.04	5.36 × 10^−47^
ribonucleoside binding	−2.04	5.36 × 10^−47^
purine ribonucleoside triphosphate binding	−2.04	5.36 × 10^−47^

**Table 4:** Up to the top 10 leaf GO terms with a *q*-value ≤ 0.01 for positive and negative log-fold changes comparing ocean water samples from BSt (Surface) to CS (Bottom) from May et al. Shown are the name of the GO term, the log-fold change from surface to bottom samples, and the *q*-value resulting from the Benjamini-Hochberg adjustment.

**Biological Process**					
**Higher in Ocean Surface Water**			**Higher in Ocean Bottom Water**		
**GO Term**	**log Change**	***q*-Value**	**GO Term**	**log Change**	***q*-Value**
d-xylose transport	−6.09	3.49 × 10^−73^	protein refolding	1.66	1.01 × 10^−167^
translation	−0.77	2.43 × 10^−57^	chromosome condensation	1.52	3.69 × 10^−50^
translational elongation	−1.11	9.60 × 10^−26^	DNA repair	1.61	1.82 × 10^−7^
transcription anti-termination	−2.79	5.66 × 10^−8^	dephosphorylation	2.04	6.62 × 10^−7^
fatty acid biosynthetic process	−1.21	6.42 × 10^−8^	de novo’ pyrimidine nucleobase biosynthetic process	3.74	4.53 × 10^−5^
GTP biosynthetic process	−5.12	7.30 × 10^−8^	RNA phosphodiester bond hydrolysis, exonucleolytic	1	5.52 × 10^−5^
UTP biosynthetic process	−5.12	7.30 × 10^−8^	mRNA catabolic process	0.93	1.69 × 10^−4^
CTP biosynthetic process	−5.12	7.30 × 10^−8^	7,8-dihydroneopterin 3′-triphosphate biosynthetic process	3.09	4.48 × 10^−3^
tricarboxylic acid cycle	−3.84	4.41 × 10^−8^	response to cadmium ion	1.45	7.42 × 10^−3^
cell division	−1.07	1.18 × 10^−5^			
**Molecular Function**					
**Higher in Ocean Surface Water**			**Higher in Ocean Bottom Water**		
**GO Term**	**log Change**	***q*-Value**	**GO Term**	**log Change**	***q*-Value**
monosaccharide binding	−6.07	6.71 × 10^−72^	histidine ammonia-lyase activity	4.54	1.93 × 10^−113^
receptor activity	−1.03	5.77 × 10^−65^	unfolded protein binding	0.83	2.85 × 10^−57^
structural constituent of ribosome	−0.68	1.12 × 10^−34^	nitrate reductase activity	7.35	4.13 × 10^−35^
DNA-directed RNA polymerase activity	−1.68	4.06 × 10^−34^	heme binding	3.38	4.23 × 10^−35^
translation elongation factor activity	−1.09	7.50 × 10^−25^	ATP binding	0.51	6.87 × 10^−34^
GTP binding	−0.92	6.40 × 10^−17^	4 iron, 4 sulfur cluster binding	2.33	3.28 × 10^−27^
GTPase activity	−0.92	8.64 × 10^−7^	prephenate dehydratase activity	3.94	4.38 × 10^−13^
nucleoside diphosphate kinase activity	−5.11	1.28 × 10^−7^	selenium binding	2	8.97 × 10^−13^
tRNA binding	−0.87	3.00 × 10^−6^	4-phytase activity	3.45	7.83 × 10^−73^
acetyl-CoA carboxylase activity	−3.91	3.46 × 10^−6^	formate dehydrogenase (NAD+) activity	1.48	9.79 × 10^−6^
**Cellular Component**					
**Higher in Ocean Surface Water**			**Higher in Ocean Bottom Water**		
**GO Term**	**log Change**	***q*-Value**	**GO Term**	**log Change**	***q*-Value**
cell outer membrane	−0.98	1.17 × 10^−43^	cytoplasm	0.74	8.53 × 10^−84^
intracellular	−0.71	1.09 × 10^−36^	bacterial-type flagellum filament	3.49	1.47 × 10^−15^
ribosome	−0.64	1.78 × 10^−33^	bacterial-type flagellum	2.01	1.53 × 10^−8^
integral component of membrane	−0.98	9.77 × 10^−24^	unknown cellular component	0.07	1.04 × 10^−6^
thylakoid	−2.08	1.07 × 10^−11^	ATP-binding cassette (ABC) transporter complex	0.6	1.04 × 10^−5^
large ribosomal subunit	−1.21	1.54 × 10^−11^	cytosolic small ribosomal subunit	3.66	9.19 × 10^−3^
acetyl-CoA carboxylase complex	−3.84	4.22 × 10^−6^			
plasma membrane	−0.29	3.37 × 10^−4^			
pyruvate dehydrogenase complex	−3.99	7.77 × 10^−4^			
proton-transporting ATP synthase complex, catalytic core F(1)	−0.37	1.36 × 10^−3^			
